# Characterization of the microDNA through the response to chemotherapeutics in lymphoblastoid cell lines

**DOI:** 10.1371/journal.pone.0184365

**Published:** 2017-09-06

**Authors:** Pamela Mehanna, Vincent Gagné, Mathieu Lajoie, Jean-François Spinella, Pascal St-Onge, Daniel Sinnett, Ivan Brukner, Maja Krajinovic

**Affiliations:** 1 CHU Sainte-Justine Research Center, University of Montreal, Montreal, Qc, Canada; 2 Department of Pediatrics, Faculty of Medicine, University of Montreal, Montreal, Qc, Canada; 3 Molecular Diagnostics Laboratory, Jewish General Hospital, McGill University, Montreal, Montreal, Qc, Canada; 4 Department of Pharmacology and Physiology, Faculty of Medicine, University of Montreal, Montreal, Qc, Canada; Wayne State University, UNITED STATES

## Abstract

Recently, a new class of extrachromosomal circular DNA, called microDNA, was identified. They are on average 100 to 400 bp long and are derived from unique non-repetitive genomic regions with high gene density. MicroDNAs are thought to arise from DNA breaks associated with RNA metabolism or replication slippage. Given the paucity of information on this entirely novel phenomenon, we aimed to get an additional insight into microDNA features by performing the microDNA analysis in 20 independent human lymphoblastoid cell lines (LCLs) prior and after treatment with chemotherapeutic drugs. The results showed non-random genesis of microDNA clusters from the active regions of the genome. The size periodicity of 190 bp was observed, which matches DNA fragmentation typical for apoptotic cells. The chemotherapeutic drug-induced apoptosis of LCLs increased both number and size of clusters further suggesting that part of microDNAs could result from the programmed cell death. Interestingly, proportion of identified microDNA sequences has common loci of origin when compared between cell line experiments. While compatible with the original observation that microDNAs originate from a normal physiological process, obtained results imply complementary source of its production. Furthermore, non-random genesis of microDNAs depicted by redundancy between samples makes these entities possible candidates for new biomarker generation.

## Introduction

Extrachromosomal nuclear circular DNAs (eccDNAs) have been known for almost 4 decades and have been characterized in several eukaryotic organisms including humans. These entities are homologous to chromosomal DNA and are heterogeneous in terms of size (<1 Kb to >1 Mb), sequence complexity and regions of origin [1]. In plants, eccDNAs are a potential intermediate in processes driving satellite repeat evolution [2] while in yeasts, eccDNAs seem to derive from ribosomal or telomeric DNA [3–4].

Until recently, most of human eccDNAs were thought to originate from intra-chromosomal homologous recombination of chromosomal tandem repeats (coding genes and satellite DNA) [1]. In 2012, Shibata and colleagues discovered a new class of eccDNA (called microDNAs) in mouse and human cells that harbor distinct features than previously characterized entities [5], suggesting different production mechanisms. These microDNAs were shorter than other eccDNAs with the majority ranging from 100 to 400 bp, showed the length periodicity and derived from unique non-repetitive genomic regions. They showed high gene density and had a high GC content. Further research from the same group suggested that at least fraction of these eccDNAs could arise from replication slippage and the mismatch repair pathway [6].

Based on the observed periodicity of size peaks of microDNAs along with the fact that DNA laddering is apoptosis hallmark [7], it is possible that a part of this new class of nucleic acids could derive through processes preceding the programmed cell death.

In this study, we extracted and sequenced microDNAs from untreated human lymphoblastoid cell lines (LCLs), or following treatment using chemotherapeutic drugs inducing apoptosis and DNA breaks such as methotrexate (MTX) and L-asparaginase (ASP) [8–9]. We identified a periodicity of 190 bp corresponding to the laddering pattern of degraded DNA reminiscent of apoptosis [10]. We also observed an influence of chemotherapeutic drug-induced apoptosis on microDNA size, diversity and redundancy and a modulation of this effect according to the sensitivity status of cells. Overall, our findings suggest that microDNAs may reflect a particular status of the cell chromatin prior to cell death.

## Material and methods

### Study samples

Cell lines were selected from human EBV immortalized Lymphoblastoid Cell Lines (LCLs) obtained from different healthy individuals and purchased from the Coriell Institute for medical research (Camden, NJ, USA), based on their sensitivity phenotype to two major chemotherapeutic drugs, methotrexate (MTX) and L-asparaginase (ASP) (**S1 Table**), as defined in a prior independent experiment, by measuring their half maximal inhibitory concentrations (IC50). Cell lines that are either intrinsically sensitive, or resistant to MTX and ASP were selected for this experiment (5 cell lines for each phenotype per drug, 20 different cell lines in total). **S1 Fig** shows a graphical representation of the drug group segregation design. MicroDNA was extracted from each cell line prior and after 48h (for ASP) and 72h (for MTX) long treatment resulting in overall 40 tested conditions distributed across 8 drug groups (4 per each drug). LCLs were cultured at 37°C and 5% CO2 in RPMI medium supplemented with 15% fetal bovine serum and antibiotics (100 IU/ml penicillin; 100 μg/ml streptomycin).

#### MicroDNA extraction from LCL nuclei

Nuclei were extracted from 1x10^7^-10^8^ cells/well according to the Nuclei Pure Prep Nuclei Isolation Kit protocol (Sigma-Aldrich NUC-201). Samples were layered on a 1.8M sucrose cushion concentration (30 ml per 10 ml homogenized samples) and spun at 30000g for 45 min at 4°C (Beckman Optima L-90K Ultracentrifuge, SW32 rotor). Supernatant were discarded and pellet were resuspended in 200 μl Nuclei PURE storage buffer [5]. MicroDNA was isolated using the Qiagen High Speed Midi Plasmid Purification Kit according to the manufacturer instructions. DNA was eluted in 1 ml of Tris-EDTA (TE) buffer solution and concentrated by adding 20 μg of glycogen, 0.1 volume of 3M sodium acetate, pH 5.2 and 2 volume of Isopropanol followed by centrifugation at 16000g for 20 minutes. The pellet was resuspended in 20 μl 1X TE and concentration determined using the Nanodrop. 1 μg was digested with the Exonuclease VII (Epicentre) and an ATP-Dependant DNase (Epicentre) to remove linear ds and ssDNA, and purified after each digestion step with MinElute.

#### MicroDNA amplification

MicroDNA elute was used for rolling circle amplification using degenerative 6-mers and Phi 29 polymerase. An in-house protocol using oligonucleotides containing internal C3 spacers which minimized production of self-priming products was used [11]. Amplified concatemers of microDNA was purified using Amicon Ultra-0.5 centrifugal filter devices then quantified using the dsDNA BR Assay Kits (Life Technologies Q32853). 1 μg of microDNA was physically sheared using Covaris S2 (10% duty cycles, intensity of 5,200 cycles per burst and a time of 180 seconds) to obtain size peaks centered around 200 bp. Sheared DNA was purified with AMPure beads and quantified. 1 μl of purified microDNA was loaded on an Agilent DNA 1000 chip to assess the quality of sheared DNA. A detailed protocol can be found at https://www.protocols.io/private/503c28af82eafa3f29140a12136767a8

#### MicroDNA sequencing

Collected microDNA material was sequenced using on the Ion Torrent™ Personal Genome Machine® (PGM, Ion 318™ Chip v2, Life Technologies) at the Integrated Clinical Genomics Center In Pediatrics at the CHU Sainte-Justine according to the manufacturer’s protocol (single-end (SE): 200 bp). Obtained reads (mean coverage 42X, 65% average of mapped reads, **S2 Table**) were mapped on the hg19 human reference genome with STAR, the ultrafast universal RNA-seq aligner (version 2.4.0) using parameter–*chimSegmentMin* to insure the *chimeric*.*junction*.*out* output file (**S2 Fig**) [12]. The obtained sequences are deposited to a public repository (BioProject database; ID, PRJNA394111; http://www.ncbi.nlm.nih.gov/bioproject/394111).

#### Cluster identification and analysis

Outputted chimeric.junction.out files were used as input for an in-house algorithm allowing the microDNA cluster identification. Coordinates (start and end) of each identified intrachromosomal chimeric junction were recursively extended by 10 bp upstream and downstream to form a cluster supporting the breakpoint consistent with a circular molecule. Extension was pursued until no new read supporting the breakpoint could be added to the cluster. **S3 Fig** offers a graphical representation of the algorithm. This “*chimeric junction method*” was compared and preferred to an adapted version of the previously published “*island method*” [5]. Briefly, using the latter method, to be considered as belonging to a putative microDNA candidate, a sequence had to match the reference genome, to be enclosed with identical upstream and downstream soft-clips and to present a size within a range of 20 to 2000 bp, slightly larger size interval than previously described to add leeway for the analysis [5–6].

MicroDNAs resulting from this analysis were organized for each drug in 4 distinct groups according to LCL phenotypes and treatment: sensitive-treated (S_T), sensitive-non-treated (S_NT), resistant-treated (R_T) and resistant-non-treated (R_NT).

#### Genomic annotation, regions enrichment analysis and cluster redundancy

Using an in-house script (provided in **S1 Appendix**;), genomic regions matching microDNA coordinates were queried against i) the RefSeq database (downloaded from UCSC server [13]) to annotate 3’UTR, 5’UTR, intronic and exonic regions. The RefSeq database was also used to annotate promoters (defined as 2kb upstream of the transcription start site (TSS) which were obtained from Ensembl75), ii) the Encode Consortium [14] to annotate open chromatin regions (GM12878 LCL was used as reference), iii) the “repeatMasker” track [15] to identify regions matching repeat elements (LINE, SINE & LTR), iv) GM12878 LCL data of the Roadmap Epigenomics Project (narrow peaks from epigenome E116) [16] to extrapolate local histone marks (H3K9ac, H3K27me3 and H3K9me3) and iv) Ensembl75 annotations to obtain TSS of expressed genes. To avoid bias caused by different microDNA size distributions, distance to TSSs was computed from the center of each microDNA. For other annotations, a minimum overlap of 1 bp was considered. Distribution graphs were generated using the R GenomicRanges package [17]. The enrichment ratio for TSS, histone marks and repeat elements was computed by comparing the number of features overlapping the regions of interest with the number of features overlapping flanking regions of the same size (at 5 kb distance) and the difference was calculated using a binomial test.

The observed distribution of microDNAs in diverse genomic regions was compared to a simulated random distribution of equivalent entities. The latter was created using the BEDTools *shuffle* function [18]; for each microDNA coordinate, a random new position was created with the same length as the original one. This step was repeated 1000 times for each original microDNA. The median was calculated and taken as the expected value. The fold ratio was inputted as the observed by expected ratio.

BEDTools' tool nuc [18] combined with an in-house algorithm (data extraction and output file manipulation, **S2 Appendix**) was used to determine the %GC of each identified microDNA and its 1000 bp flanking regions.

MicroDNA cluster intersection was determined using an in-house script (data extraction and file parsing; **S3 Appendix**) exploiting BEDTools Intersect function [18]. Sequence reads that overlap between samples at least 1 bp, and are part of continual genomic segment, are referred as “cluster intersects”. Sequence reads that originate from the same gene, with or without overlapping sequence position (no need for “continuality”), are referred using term “gene intersects”.

Statistical significance was computed with the R Project for Statistical Computing [19]. When appropriate, Mann-Whitney-U, Wilcoxon signed rank tests, Fisher's Exact Test or Chi-Square were used for data comparison.

## Results

MicroDNAs were isolated from 40 LCL samples (20 different LCLs before and after treatment with MTX and ASP) divided into equal groups according to the drug received (MTX, ASP or none) and their sensitivity status (S1 Table). MicroDNAs were sequenced and aligned on the reference human genome to identify their loci of origin.

### MicroDNAs' loci of origin are not random

**Fig 1:**
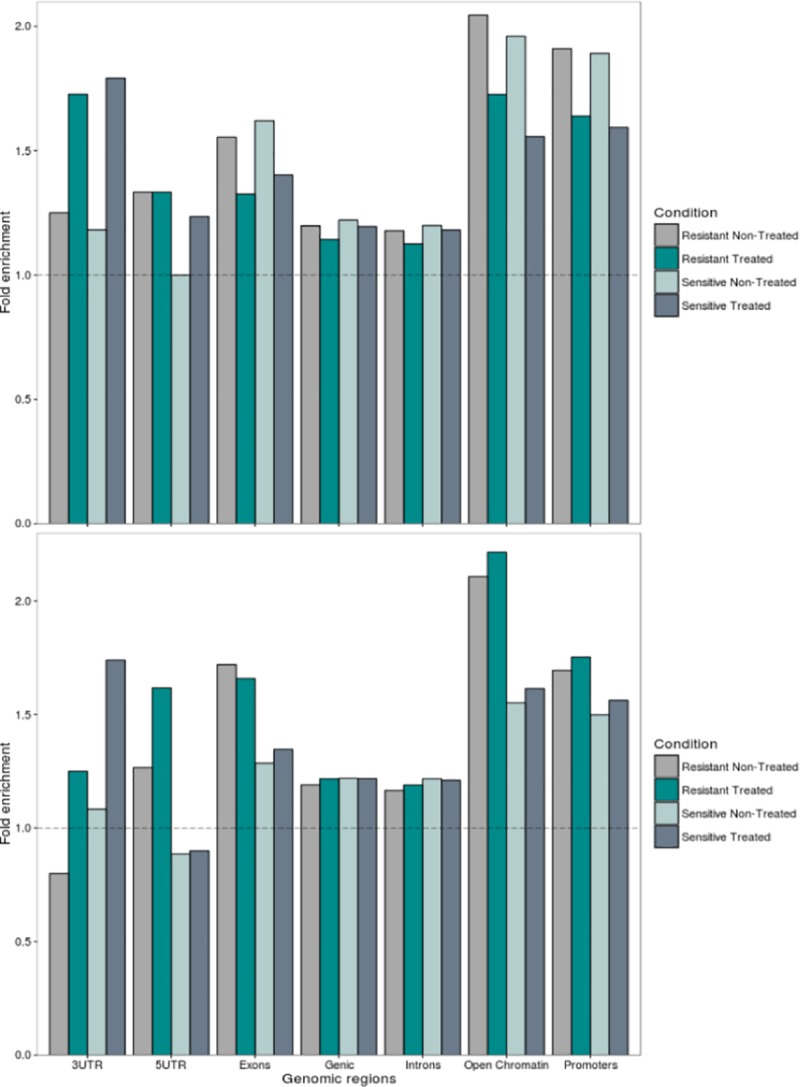
MicroDNA are significantly enriched in coding and active genomic regions. Fold enrichment is calculated as the ratio of the observed by expected number. Expected numbers were computed by generating 1000 random new positions with lengths corresponding to those of identified microDNAs and outputting the median. The dotted line shows a hypothetical situation where expected number would be equal to the observed number. **Top**: Methotrexate (MTX). **Bottom**: Asparaginase (ASP). Statistical significance was assessed using Fisher's Exact test (S3 Table).

Regarding overall distribution of gene regions, in all studied drug groups, intronic regions were predominant, followed by exons and promoters with lowest representations of UTRs (**Table 1**). Equilibrium between genomic segments belonging to “open” versus “closed” chromatin is dynamic, but vast majority of the genome is structurally “closed” at any time, explaining why fewer microDNA in total were mapped to open chromatin (**Table 1**). The partitioning among these chromatin fractions could indicate microDNA genesis; to assess the stochasticity of their genomic distribution, a simulated random distribution of equivalent entities was performed and compared to the observed one. A significant enrichment was noted for most of the functional regions compared to random distribution in all LCLs regardless of treatment and their sensitivity status (p < 2.2e-16, **Fig 1**, **S3 Table**). A stronger enrichment was identified for exons and promoters, compared to introns (mean fold enrichment of 1.49 for exons, 1.86 for promoters and 1.85 for “active” regions *vs*. 1.18 for introns, Fig 1, S3 Table). Importantly, higher frequency of microDNAs originating from exons and open chromatin regions were detected in treated then in non-treated cells (7.7% *vs*. 6.7% and 9.1% *vs*. 7.9% respectively, Table 1, p < 0.0001).

**Table 1:** Genomic distribution of microDNAs per drug and per condition.

Drug	Condition	Genic	Exonic	Intronic	5'UTR	3'UTR	Promoter	NA	Active	Inactive	Total
ASP	R_T	10493	1519	9789	38	20	468	9342	1958	18463	20421
	R_NT	6709	910	6264	19	8	305	6272	1075	12285	13360
	S_T	12453	1934	11857	27	40	464	11122	2272	21942	24214
	S_NT	14944	1891	14265	31	26	559	13367	2202	26841	29043
MTX	R_T	6103	945	5750	20	19	259	6195	1214	11441	12655
	R_NT	2937	356	2754	8	5	149	2703	452	5362	5814
	S_T	13697	2074	12959	42	43	545	12738	2222	24931	27153
	S_NT	7265	1055	6814	17	13	348	6401	1231	12865	14096

ASP: Asparaginase; MTX: Methotrexate; R_T: Resistant and treated; R_NT: Resistant and non-treated; S_T: Sensitive and treated; S_NT: Sensitive and non-treated; NA: Number of microDNAs that mapped on regions that we did not investigate (e.g., intergenic regions).

**Fig 2:**
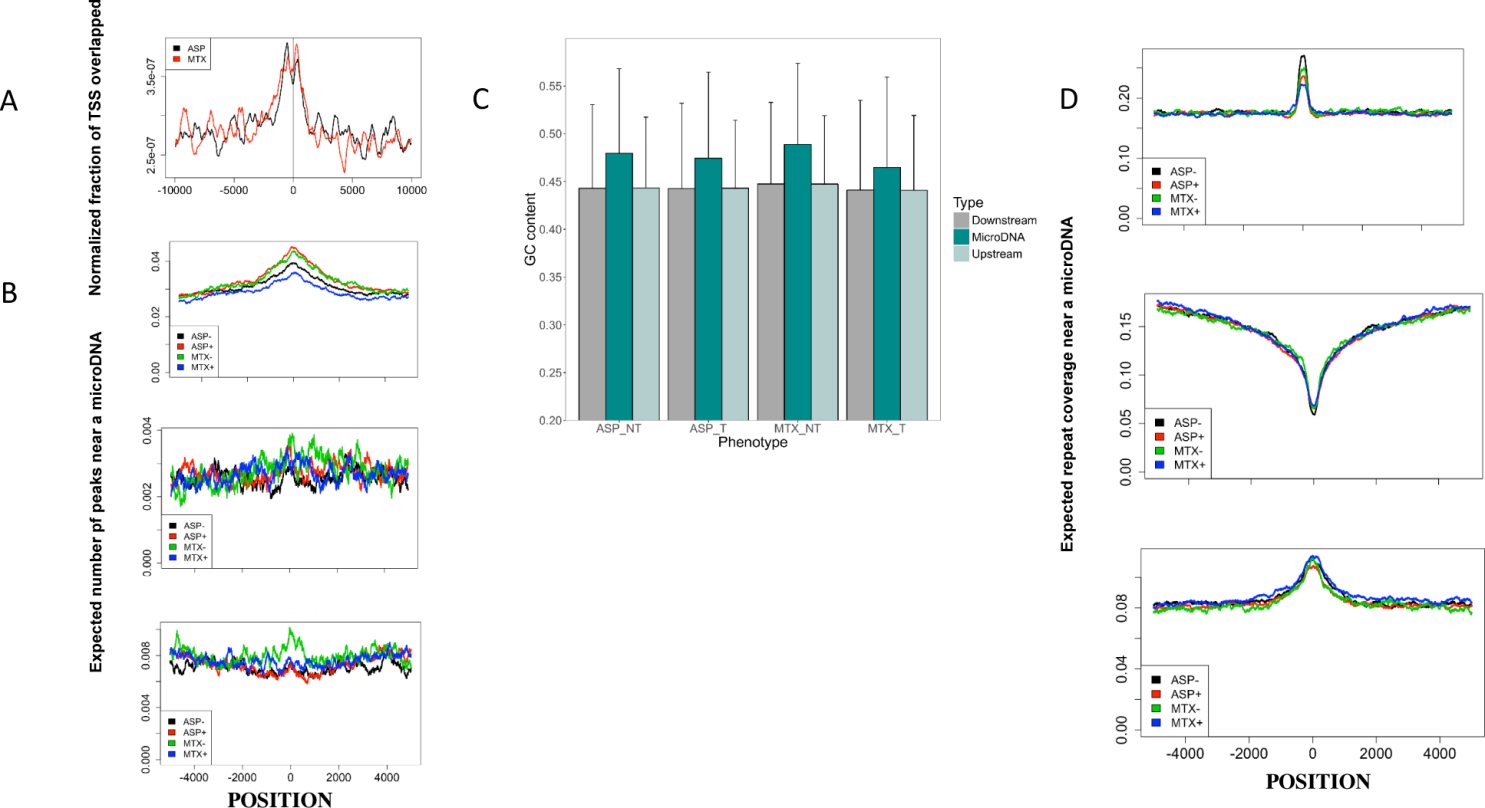
MicroDNAs’ loci of origin are not random. Negative values on the x-axis correspond to the left of the microDNA centers and positive values to their right, according to genomic coordinates for (A) (B) and (C) (strandedness not considered). (**A**) Distribution of microDNAs near transcription start sites (TSSs) of genes expressed at more than 1 FPKM (fragments per kilobase of transcript per million mapped reads) in the GM12878 lymphoblastoid cell line. The y axis corresponds to the fraction of TSS-centered windows overlapped by a microDNA at any given position, normalized by the total number of microDNAs, per drug (ASP: Asparaginase, MTX: Methotrexate). (**B**) Distribution of histone marks in the GM12878 cell line, with respect to the locations of microDNAs identified in tested LCLs per drug and per treatment. The y axis can be interpreted as the expected number of peaks at any given position near a microDNA. **Top**: Transcriptional activation-related histone mark H3K9ac. **Middle**: Histone mark associated with inactive gene promoters, H3K27me3. **Bottom**: Gene silencer histone mark H3K9me3. (**C**) GC content of identified microDNAs *vs*. their 1000 bp flanking regions (FR), per drug and per condition. Statistical significance was assessed using Mann-Whitney tests (p < 2.2e-16***; T *vs*. FR and NT *vs*. FR, for both drugs). (**D**) Genomic distribution of 3 classes of repeated elements with respect to the locations of microDNAs identified in tested cell lines, per drug and per treatment. **Top**: Short interspersed elements (SINEs). **Middle**: Long interspersed elements (LINEs). **Bottom**: Long terminal repeats (LTRs). Significance enrichment (p<0.0001, estimated by binomial probability) is found for the data depicted in A, B and D (top panel).

MicroDNAs were located close to TSSs (**Fig 2A**, p < 0.0001), they were enriched in transcribed regions (close to H3K9ac peaks, **Fig 2B**, p < 0.0001, binomial test) whereas, no difference in repressed regions (harboring H3K27me3 and H3K9me3 histone marks) was noted (**Fig 2B**). Furthermore, microDNAs presented a mean GC content of 47.7% (**Fig 2C**) which was significantly higher than the content of their 1000 bp flanking regions (p < 2.2e-16). The pattern was similar regardless of the drug, treatment or sensitivity group (**S4 Fig**). By exploring genomic distribution of different classes of repeat elements, we identified an enrichment of microDNAs near short interspersed nuclear elements (SINEs) and long terminal repeats (LTRs), but less frequently positioned near long interspersed nuclear elements (LINEs) (**Fig 2D**, p < 0.0001).

In accordance with previous reports [5–6], general feature patterns suggested a non-random distribution of microDNA loci and a biased origin towards open regions of the chromatin. This pattern was similar across all studied drug groups.

### Drug-induced apoptosis modulates microDNA size and diversity

**Fig 3:**
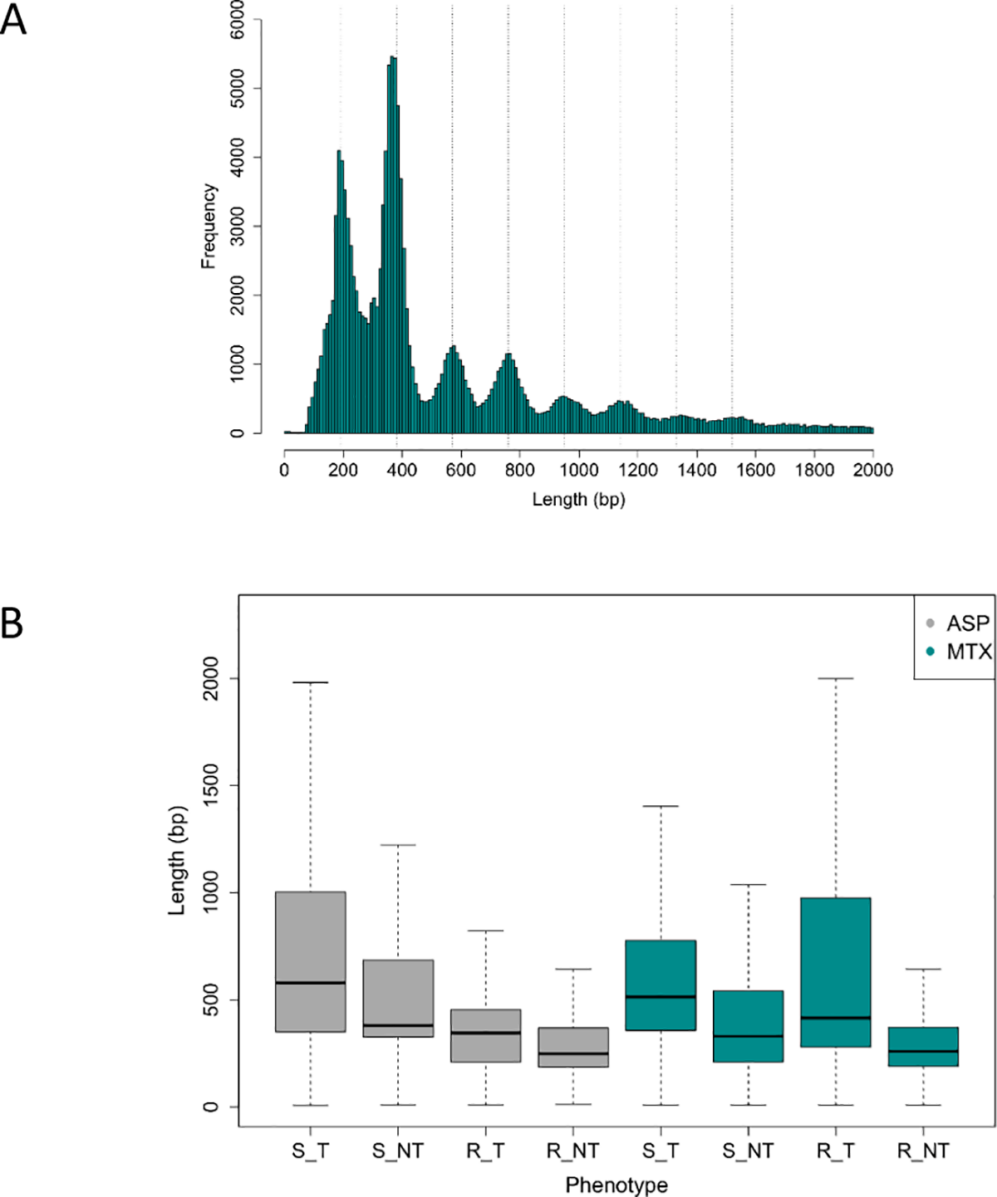
MicroDNA length and periodicity. Size distribution in base pairs (bp) of all identified microDNAs (**A**) regardless of drug used for treatment. Vertical lines depict the 190 bp periodicity. (**B**) per drug (ASP: Asparaginase, MTX: Methotrexate) and per sensitivity status (resistant: R, sensitive: S, treated: T, non-treated: NT).

On average 3669 unique microDNA clusters were found per sample. Matching previous results [5–6], a general mean length of 379 bp (95% CI = 152–1465 bp, IQR range 256 bp-729 bp) with 2 peaks at ~200 bp and ~400 bp was observed (**Fig 3A**). Furthermore, we identified a peak periodicity of ~190 bp.

MicroDNAs’ mean length was dependent on treatment and sensitivity status (**Fig 3B**). Cell lines treated with either ASP or MTX showed on average significantly longer microDNAs than their non-treated counterparts (p < 2.2e-16, S_T vs. S_NT & R_T vs. R_NT). Size distribution was further modulated by the sensitivity status of cell lines with a significantly increased length noted for sensitive cells (p < 2.2e-16, S_T vs. R_T treated with ASP and p = 5.626e-10, S_T vs. R_T treated with MTX).

**Fig 4:**
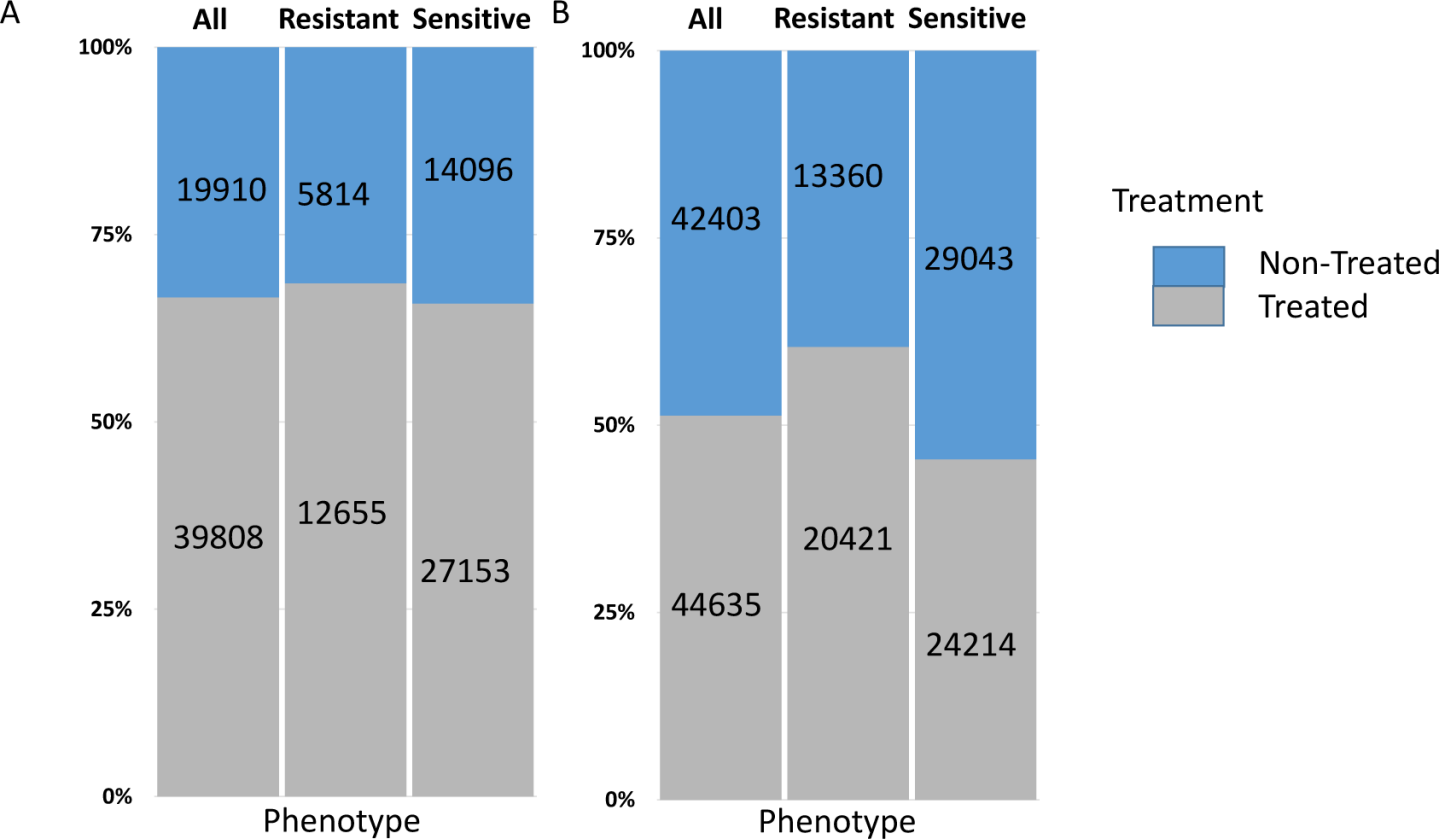
MicroDNA generation in relation to treatment. Percentage (%) of unique microDNAs generated from LCL samples when treated *vs*. non-treated with (**A**) Methotrexate (MTX) or (**B**) Asparaginase (ASP). Treated *vs*. non-treated samples: **Left**, regardless of sensitivity/resistance status; **Center**, in resistant cells; **Right**, in sensitive cells. Numbers on the graph represent the number of unique microDNAs generated in each group.

For each condition, the total number of unique microDNAs was also assessed across groups. MTX treated cells generated higher numbers of unique microDNA entities (**Fig 4A**), either when comparing all treated to non-treated cells (66.7% of microDNAs originated from T samples vs. 33.3% from NT samples, p < 0.0001), or when effect of treatment was assessed within resistant (68.5% from T samples vs. 31.5% from NT, p < 0.0001) or sensitive phenotype (65.8% from T samples vs. 34.2% from NT, p < 0.0001). This effect was less obvious in ASP treated cells (**Fig 4B**). Irrespective of treatment, sensitive cells generated higher number of microDNA diversity than resistant cells (Fig 4). Likewise, average number of unique microDNAs differed by the treatment and sensitivity status (**S5 Fig**). Altogether, these results suggested that microDNA diversity is modulated by treatment-induced apoptosis.

### MicroDNAs sequence motifs are redundant between LCLs

**Fig 5:**
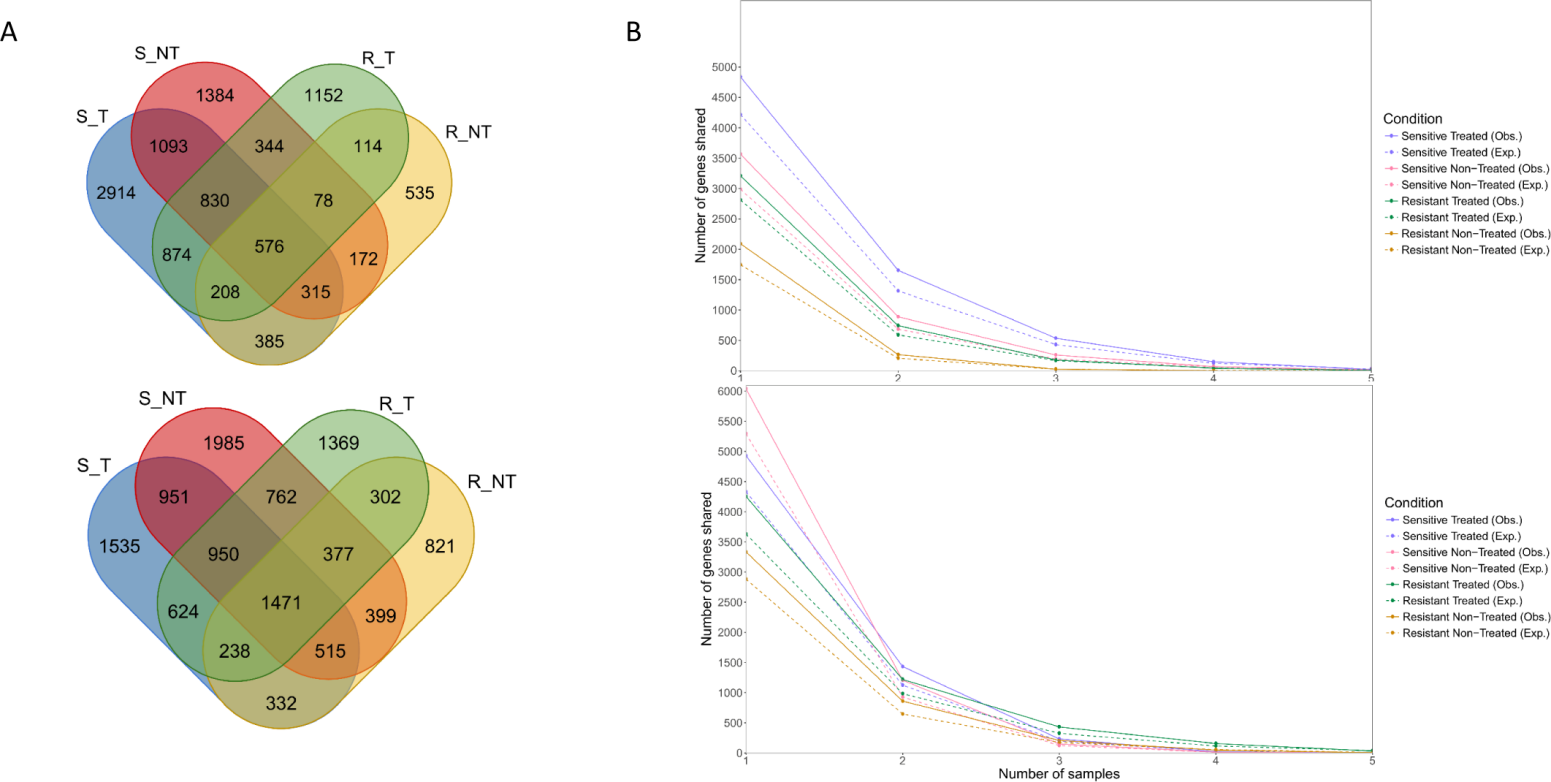
Some microDNAs are shared between and within drug groups. Number of genes where from microDNA derived shared between drug groups (Sensitive: S; Resistant: R; Treated: T; Non-Treated: NT) (**A**) Observed numbers per drug **Top**: MTX **Bottom**: ASP (**B**) Observed *vs*. expected numbers of microDNA-derived genes per group. Expected numbers were computed by generating 1000 random new microDNAs with lengths corresponding to those we identified. **Top**: MTX **Bottom**: ASP. p<0.01 (estimated by chi-square) for the difference between observed and expected numbers in all cases except MTX R_NT group.

To examine redundancy of microDNAs between samples, we analyzed whether microDNAs originating from same genomic loci were shared between two or more samples. Two types of intersects were analyzed, cluster intersects, referring to microDNAs that overlap between samples and gene intersects, referring to microDNAs that originate from the same gene, with or without overlapping sequence position. In general, the number of times that these intersects were observed between any two cell lines largely exceeds the number expected by chance, as estimated from microDNA sequence complexity compared to human genome (p < 0.0001). When intersects were compared across drug groups, a higher number of gene intersects *vs*. cluster intersects was observed in all of them (**Table 2**), whereas higher frequency of both intersects types was seen in treated than in non-treated LCLs (p<0.0001, except for cluster intersects in MTX R cells, Table 2). MTX sensitive cell lines have also higher number of intersects compared to resistant ones. A specific pattern of microDNA-derived genes was presented in **Fig 5A** showing proportion of those that overlap across drug groups and those that are unique to each of them. **S6 Fig** sheds light on the specific pattern of these genes shared between different samples of the same drug group. Furthermore, the number of observed gene intersects (in terms of gene combinations for each group) was significantly higher than that of any other possibility expected by chance (p < 0.01, except for MTX R-NT cells, **Fig 5B**).

**Table 2:** Percentage of shared entities between samples. **Left**: Gene intersects, microDNA derivied for the same gene, shared between ≥ 2 samples. **Right**: Cluster intersects, microDNA derived from the same genomic position shared between ≥ 2 samples with > = 1 bp overlap. The number/total and (%) of intersects per drug group are indicated. Difference between groups was assessed using a two-tailed Chi-square test.

Gene intersects		Cluster intersects	
N (%)	p	N (%)	p
MTX			
S_T	**< 0.0001**	S_T	**< 0.0001**
2357/7195 (32.8)		606/27153 (2.2)	
S_NT		S_NT	
1227/4792 (25.6)		153/14096 (1.1)	
R_T	**< 0.0001**	R_T	**ns**
965/4174 (23.1)		105/12655 (0.8)	
R_NT		R_NT	
291/2382 (12.2)		41/5814 (0.7)	
ASP			
S_T	**< 0.0001**	S_T	**< 0.0001**
1687/6615 (25.5)		564/24214 (2.3)	
S_NT		S_NT	
1371/7408 (18.5)		354/29043 (1.2)	
R_T	**< 0.0001**	R_T	**< 0.0001**
1840/6092 (30.2)		545/20421 (2.7)	
R_NT		R_NT	
1120/4453 (25.2)		219/13360 (1.6)	

ASP: Asparaginase; MTX: Methotrexate; R_T: Resistant and treated; R_NT: Resistant and non-treated; S_T: Sensitive and treated; S_NT: Sensitive and non-treated.

These results further support the non-random genesis of microDNAs along the genome and the modulation of their number by treatment, and in some instances, by cell sensitivity status.

## Discussion

MicroDNAs are a new type of nucleic acids originally identified by Shibata *et al*. in 2012 [5]. To further characterize this novel entity and attempt to elucidate its production process, we analyzed microDNA in intrinsically either sensitive or resistant human LCLs, before and after treatment with 2 chemotherapeutic drugs (MTX or ASP). To avoid cell line-specific effect, 10 different LCLs (originating from different individuals) for each drug have been used in the analysis.

The periodicity of 190 bp that is observed in our samples is similar to the length of degraded DNA fragments generated during apoptosis by Caspase-Activated DNase (CAD), also known as DNA fragmentation factor 40 kDa (DFF40) [10]. Cleavage of ICAD (Inhibitor of Caspase-Activated DNase) by caspase 3 results in the activation of CAD, which in turn cleaves exposed chromatin DNA at internucleosomal linker regions, producing the known laddering pattern viewable by electrophoresis after fragment separation (multiples of ~180–200 bp) [20,21]. In contrast, the fragments produced during necrosis do not usually show the same periodicity pattern, resulting instead in a smear during electrophoresis [22].

A larger number of microDNAs were produced in LCLs treated with both chemotherapeutic drugs than in their non-treated counterparts, with a more marked effect observed for MTX-treated cells. Moreover, sensitive samples showed a larger abundance of microDNAs than the resistant ones. An increase in microDNA size following drug treatment might be explained by the activation of caspase-independent execution pathways such as the one mediated by the apoptosis-inducing factor, which induces a large-scale DNA fragmentation [23]. Given that a methotrexate treatment was shown to induce a caspase 3-independent apoptosis in proliferating CD4+ T cells [24], we could speculate that the activation of an alternative apoptosis pathway in a subset of treated cells could lead to the observed shift in distribution of DNA fragments size. Together, these results suggest that microDNA is reminiscent of the apoptotic cells; it shows characteristics of apoptotic by-products by their size, and the observed treatment-induced variation in the number of generated entities corroborates the possibility of an apoptosis-modulated microDNA generation. It is worth noting that some of microDNA features differed also by sensitivity status, possibly reflecting inherent component in drug response. In line with previous observations [5–6], analysis of the genomic distribution showed enrichments of microDNAs in gene regions as well as near TSS. This analysis also revealed a high GC content in microDNAs, feature generally associated with an open chromatin configuration [25,26]. This was further confirmed by comparison to the ENCODE “open chromatin” [14] and the *Epigenome Roadmap “*H3K9ac” transcriptional activation-related histone mark [16]. Overall, these results suggested a preferential origin of microDNAs from metabolically “active” chromatin sites. We also identified enrichments near SINEs and LTRs, but less frequent position near LINEs. Non-random generation of DNA fragments during apoptosis was suggested by Di Filippo who identified GC-rich isochores as preferential targets during oxygen deprivation-induced apoptosis in eukaryotic cells producing fragments preferentially originating from genes and surrounding regions as well as from interspersed repeats [27]. The enrichment of microDNAs near SINEs and LTRs loci could also suggest the involvement of the homologous recombination pathway [28,29], although this function was previously reported as not essential for microDNA production [6]. On the other hand, the characteristic microhomology exhibited upstream and downstream of microDNAs (2 to 15 bp direct repeats [5]) was proposed as substrate of a homology-mediated circularization coupled with flap resection and ligation [6], which would allow apoptosis-derived products to form circularized DNA sequences.

While recently Dillon and colleagues proposed that microDNAs could derive from RNA metabolism [6], our results suggest that microDNA production is in part modulated by apoptosis, possibly arising through early apoptosis-driven fragmentation. Both processes (RNA metabolism and apoptosis) are part of a normal cellular physiology and participate in tissue homeostasis. Given microDNAs diversity, several origins are conceivable and further investigations will be needed to ascertain them. Importantly, given non-random positioning within the genome, notably close to transcriptionally active genes, microDNAs may reflect a specific status of the cell prior to cell death, which is even more appealing given their striking redundancy between different LCLs of the same group depicted here by treatment/sensitivity status. The fact that microDNA will have exceptional exonuclease resistance due to its circular topology, might make this class of molecules good biomarker candidates in a variety of human samples and conditions. Indeed, microDNA has been recently detected in the liquid biopsy specimen suggesting that it should be added to the repertoire of circulating nucleic acids being studied as diagnostic biomarkers[30]

## Supporting information

S1 Fig Visual representation of the segregation of samples. (DOC)

S2 Fig Analysis workflow. (DOC)

S3 Fig **(A)** Graphic representation of the chimeric junction algorithm. **(B)** Pseudo-code describing the algorithm.
(DOC)

S4 Fig GC content of identified microDNAs *vs*. their 1000 bp flanking regions (FR). (DOC)

S5 Fig Number of unique microDNAs generated from LCL samples when treated *vs*. non-treated. (DOC)

S6 Fig Number of shared microDNA gene clusters within drug groups. (DOC)

S1 Table LCL samples information. (DOC)

S2 Table Quality statistics obtained from the mapped sequencing outputs using STAR. (DOC)

S3 Table Fisher's Exact Test related p-values and fold enrichment values per drug group. (DOC)

S1 Appendix Enrichment and annotation of microDNA (enrichissement_annotation_microDNA_PlosOne.txt). (TXT)

S2 Appendix Data extraction and output file manipulation (extract_GC_PlosOne.txt). (TXT)

S3 Appendix Data extraction and file parsing (intersect_microDNA_PlosOne.txt). (TXT)
