# Supplementary material for: Characterization of the microDNA through the response to chemotherapeutics in lymphoblastoid cell lines
Source: PLoS One. 2017 Sep 6;12(9):e0184365. doi: 10.1371/journal.pone.0184365 (PMC5587290; doi:10.1371/journal.pone.0184365)
Supplement: S1 Fig — (DOC) [file pone.0184365.s001.doc]

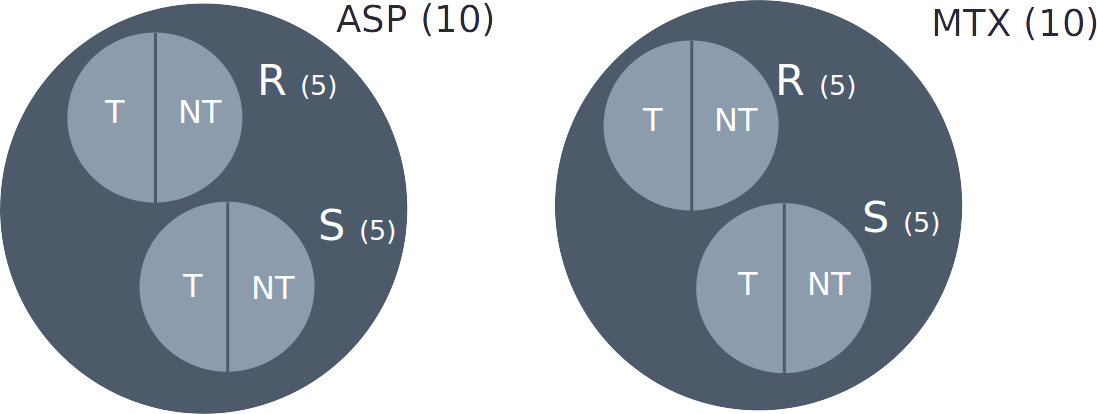


**S1 Fig Visual representation of the segregation of samples.** ASP: Asparaginase; MTX: Methotrexate; R: Resistant; S: Sensitive; T: Treated; NT: Non-Treated. The number in parenthesis represents the number of samples in each condition. N = 5 in R and S represents 5 samples in T and 5 samples in NT. See also S1 Table.
