# Supplementary material for: Characterization of the microDNA through the response to chemotherapeutics in lymphoblastoid cell lines
Source: PLoS One. 2017 Sep 6;12(9):e0184365. doi: 10.1371/journal.pone.0184365 (PMC5587290; doi:10.1371/journal.pone.0184365)
Supplement: S2 Fig — (DOC) [file pone.0184365.s002.doc]

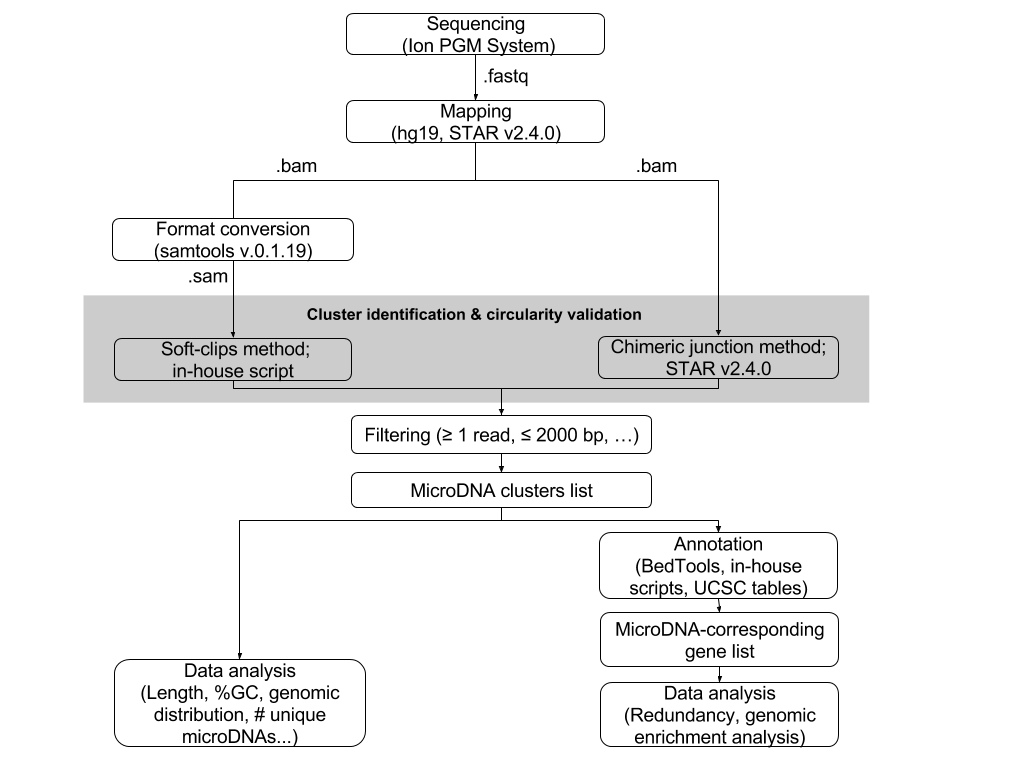


**S2 Fig Analysis workflow.** Boxes represent the analysis/filtering steps. The resulting file formats are presented above/next to the directing arrows.
