# Supplementary material for: Characterization of the microDNA through the response to chemotherapeutics in lymphoblastoid cell lines
Source: PLoS One. 2017 Sep 6;12(9):e0184365. doi: 10.1371/journal.pone.0184365 (PMC5587290; doi:10.1371/journal.pone.0184365)
Supplement: S3 Fig — (A) Graphic representation of the chimeric junction algorithm. (B) Pseudo-code describing the algorithm. (DOC) [file pone.0184365.s003.doc]

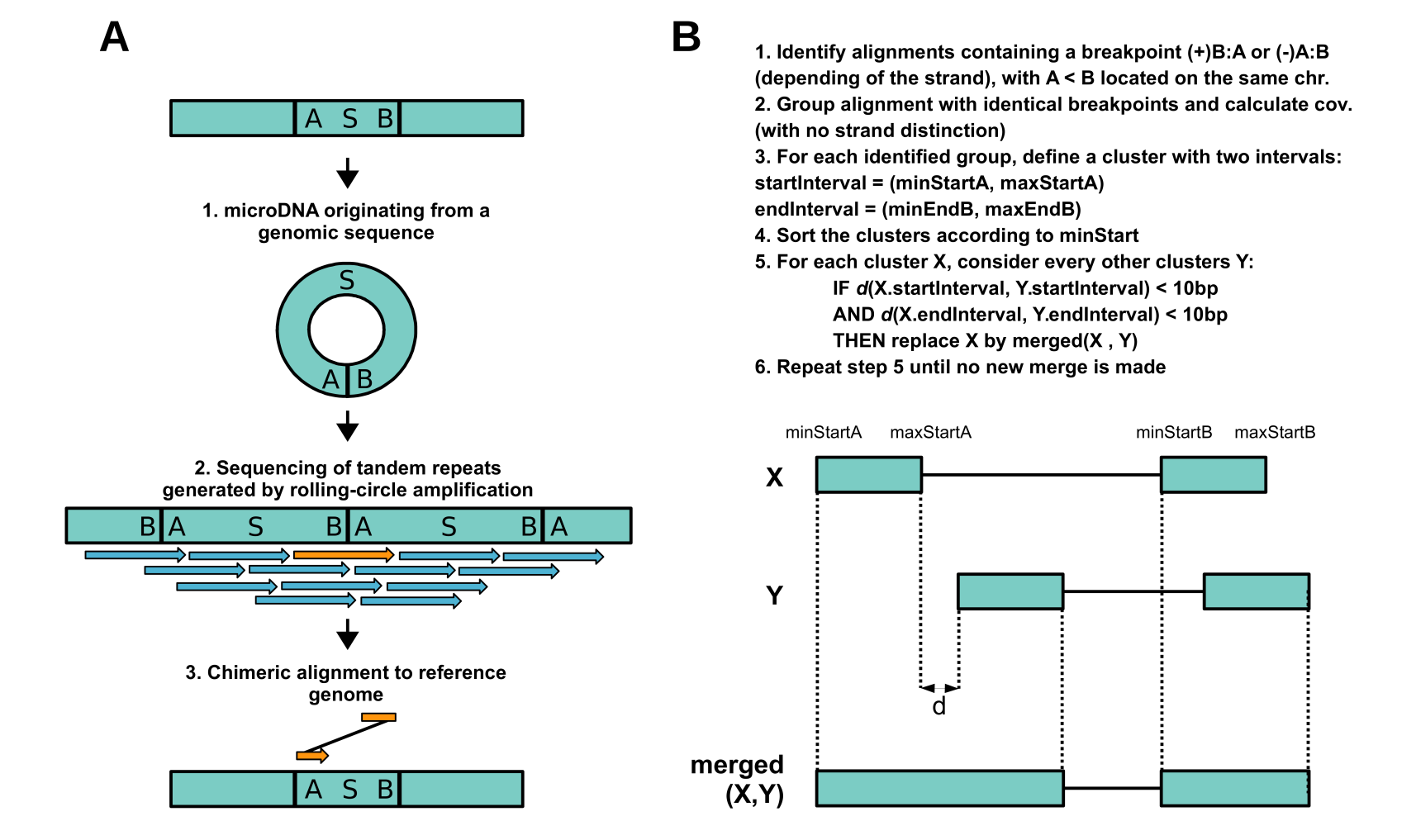
**S3 Fig (A)** Graphic representation of the chimeric junction algorithm. **(B)** Pseudo-code describing the algorithm.
