# Supplementary material for: Characterization of the microDNA through the response to chemotherapeutics in lymphoblastoid cell lines
Source: PLoS One. 2017 Sep 6;12(9):e0184365. doi: 10.1371/journal.pone.0184365 (PMC5587290; doi:10.1371/journal.pone.0184365)
Supplement: S4 Fig — (DOC) [file pone.0184365.s004.doc]

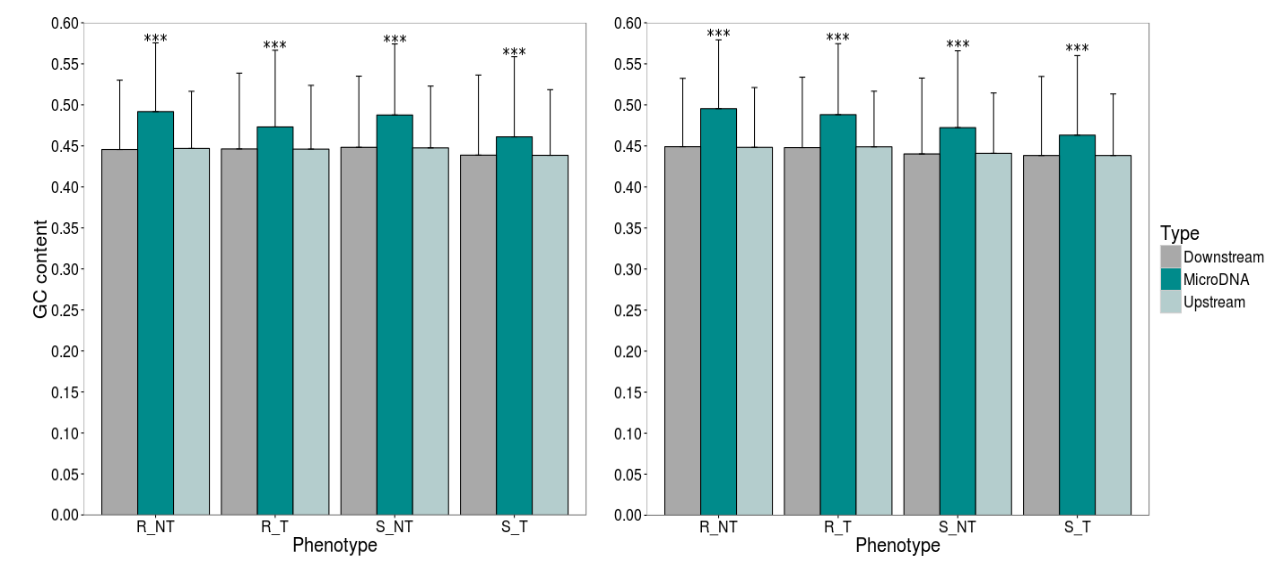


**S4 Fig GC content of identified microDNAs *vs*. their 1000 bp flanking regions (FR).** S_T: Sensitive and treated; S_NT: Sensitive and non-treated; R_T: Resistant and treated; R_NT: Resistant and non-treated. **Left:** Methotrexate used for cell treatment. **Right:** Asparaginase used for cell treatment. Statistical significance was assessed using Mann-Whitney tests (p < 2.2e-16***; R_T *vs.* FR, R_NT *vs.* FR, S_T *vs.* FR and S_NT *vs.* FR for both drugs).
