# Supplementary material for: Characterization of the microDNA through the response to chemotherapeutics in lymphoblastoid cell lines
Source: PLoS One. 2017 Sep 6;12(9):e0184365. doi: 10.1371/journal.pone.0184365 (PMC5587290; doi:10.1371/journal.pone.0184365)
Supplement: S5 Fig — (DOC) [file pone.0184365.s005.doc]

**
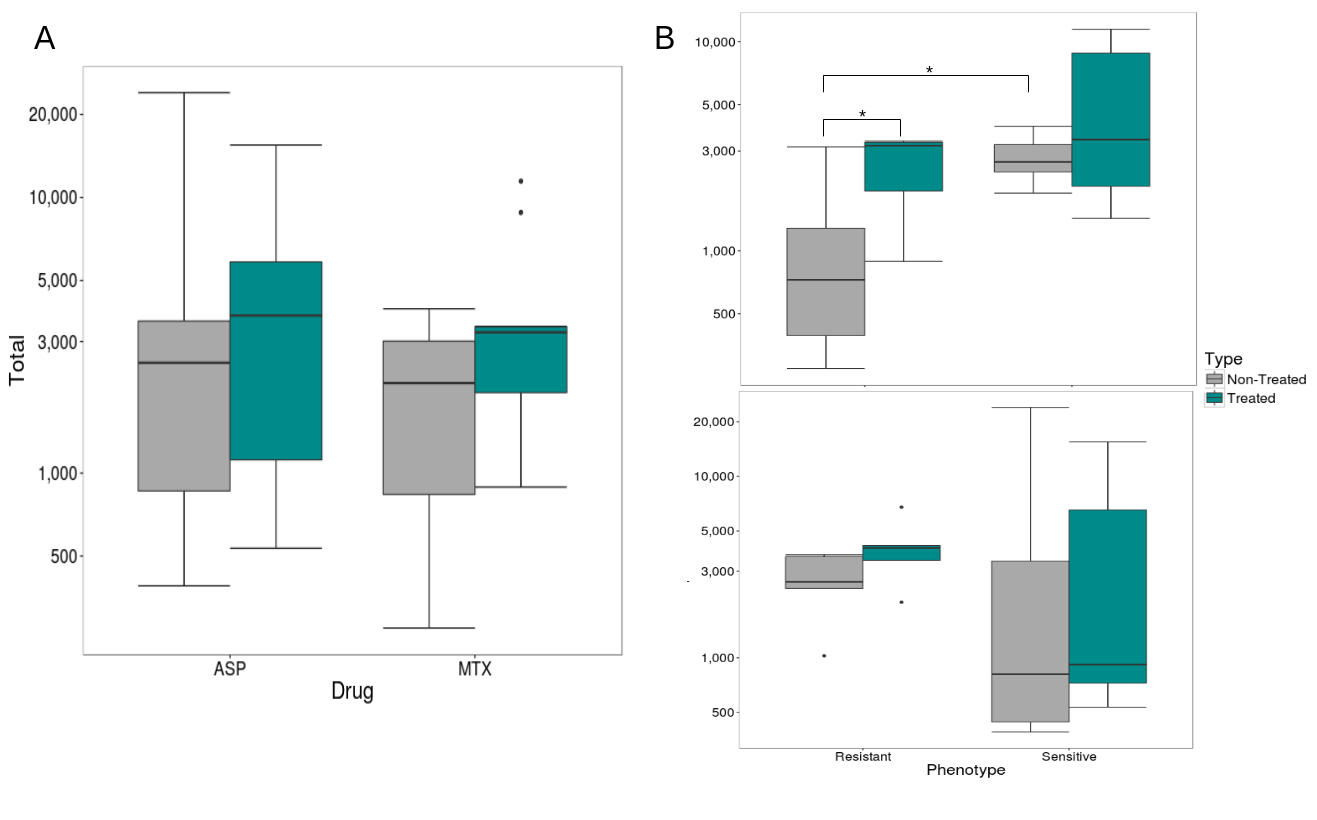
**

**S5 Fig Number of unique microDNAs generated from LCL samples** when treated *vs*. non-treated with Asparaginase (ASP) or Methotrexate (MTX) (**A**) per drug (**B**) per condition and per drug **Top:** MTX **Bottom:** ASP.

Statistical significance was computed using Mann-Whitney tests (p = 0.03* for MTX_SNT *vs*. MTX_RNT and MTX_RT *vs*. MTX_RNT) where S: Sensitive, R: Resistant, T: Treated and NT: Non-Treated).
