# Supplementary material for: Characterization of the microDNA through the response to chemotherapeutics in lymphoblastoid cell lines
Source: PLoS One. 2017 Sep 6;12(9):e0184365. doi: 10.1371/journal.pone.0184365 (PMC5587290; doi:10.1371/journal.pone.0184365)
Supplement: S6 Fig — (DOC) [file pone.0184365.s006.doc]

**
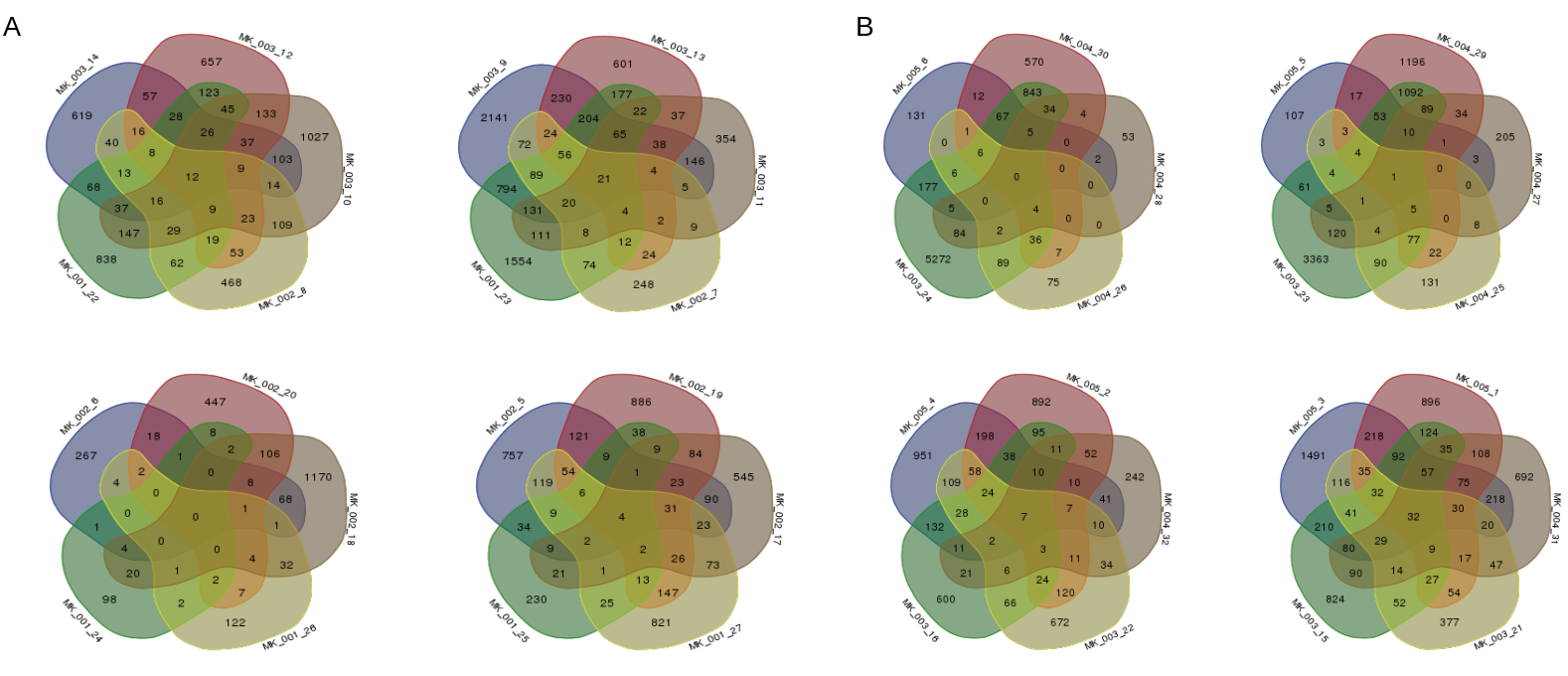
**

S_NT

S_T

R_T

R_NT

S_NT

R_T

S_T

R_NT

**
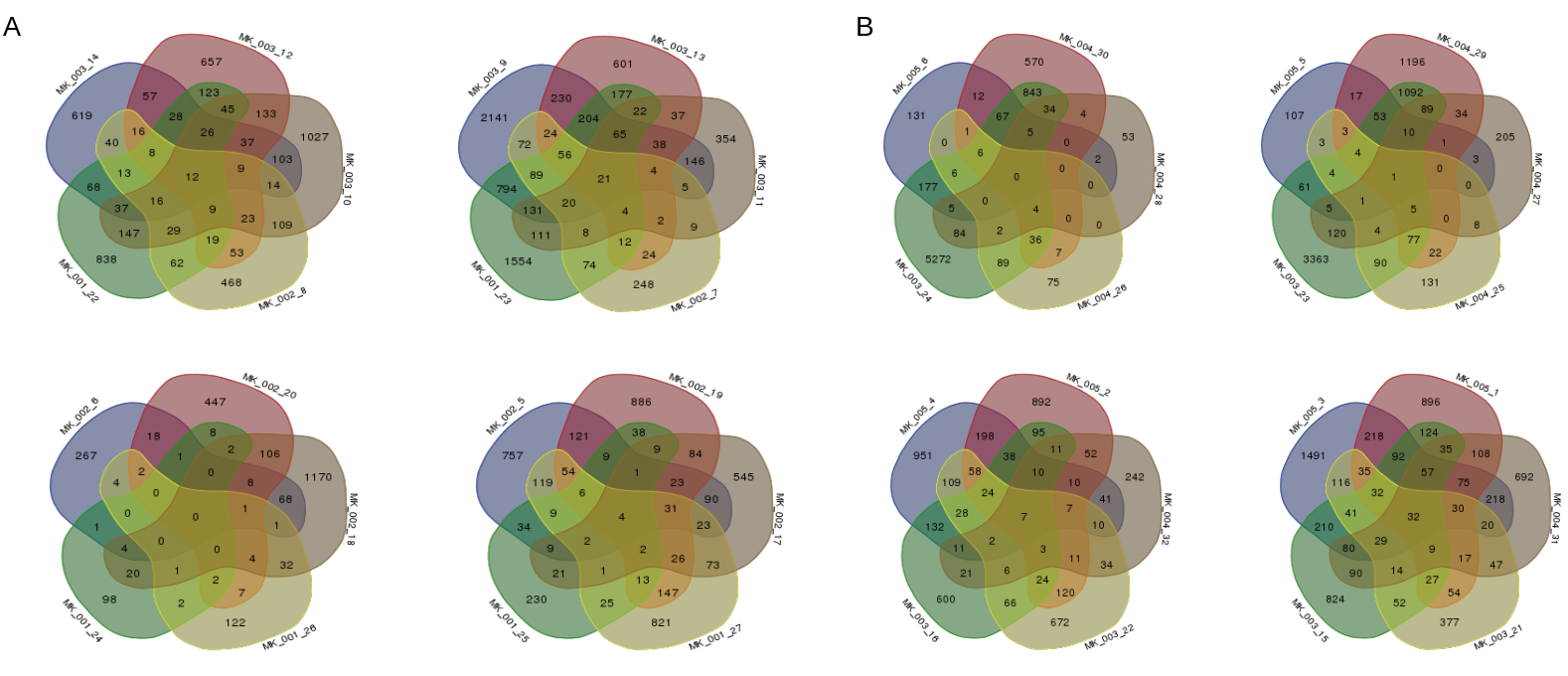
**

**S6 Fig** Number of shared microDNA gene clusters within drug groups (Sensitive: S; Resistant: R; Treated: T; Non-Treated: NT) Each group contains n=5 samples. (**A**) MTX (**B**) ASP.
