# Supplementary material for: Characterization of the microDNA through the response to chemotherapeutics in lymphoblastoid cell lines
Source: PLoS One. 2017 Sep 6;12(9):e0184365. doi: 10.1371/journal.pone.0184365 (PMC5587290; doi:10.1371/journal.pone.0184365)
Supplement: S1 Table — (DOC) [file pone.0184365.s007.doc]

**S1 Table.** **LCL samples information.**

All cell line names start with the prefix “GM” before the numbers. R: Resistant; S: Sensitive; ASP: Asparaginase; MTX: Methotrexate; +: Treated; -: Non-Treated.
