# Supplementary material for: Characterization of the microDNA through the response to chemotherapeutics in lymphoblastoid cell lines
Source: PLoS One. 2017 Sep 6;12(9):e0184365. doi: 10.1371/journal.pone.0184365 (PMC5587290; doi:10.1371/journal.pone.0184365)
Supplement: S2 Table — (DOC) [file pone.0184365.s008.doc]

**S2 Table. Quality statistics obtained from the mapped sequencing outputs using STAR.**

MTX: Methotrexate; ASP: Asparaginase; AVG: Average.
