# Supplementary material for: Characterization of the microDNA through the response to chemotherapeutics in lymphoblastoid cell lines
Source: PLoS One. 2017 Sep 6;12(9):e0184365. doi: 10.1371/journal.pone.0184365 (PMC5587290; doi:10.1371/journal.pone.0184365)
Supplement: S3 Table — (DOC) [file pone.0184365.s009.doc]

**S3 Table. Fisher's Exact Test related p-values and fold enrichment values per drug group.**

MTX: Methotrexate; ASP: Asparaginase; S_T: Sensitive and treated; S_NT: Sensitive and non-treated; R_T: Resistant and treated; R_NT: Resistant and non-treated. All p-values < 0.05 were considered statistically significant.
